# Serum uric acid predicts incident metabolic syndrome in the elderly in an analysis of the Brisighella Heart Study

**DOI:** 10.1038/s41598-018-29955-w

**Published:** 2018-08-01

**Authors:** Arrigo Francesco Giuseppe Cicero, Federica Fogacci, Marina Giovannini, Elisa Grandi, Martina Rosticci, Sergio D’Addato, Claudio Borghi

**Affiliations:** Hypertension and Atherosclerosis Research Group, Medical and Surgical Sciences Department, Sant’Orsola-Malpighi University Hospital, Via Albertoni 15, 40138 Bologna, Italy

## Abstract

Several epidemiological studies report a positive correlation between hyperuricemia and metabolic syndrome (MetS) in adults, which hyperuricemic subjects seem to more easily develop. We aimed to verify if serum uric acid (SUA) concentrations were positively associated with MetS prevalence and middle-term (4-year) incidence in older overall healthy subjects. We also purposed to identify which SUA cut-off values could be functional in MetS diagnosis in addition to the traditionally used parameters. For this reason, we selected from the historical cohort of the Brisighella Heart Study 923 older healthy subjects repeatedly visited during the 2008 and 2012 population surveys. In our sample, MetS was more frequent for higher SUA concentrations rather than the population’s mean in both men [OR = 2.12, 95%C.I.(1.55, 2.90)] and women [OR = 2.69,95%C.I.(1.91, 3.78)]. ROC analysis showed SUA was predictive of MetS in the whole population [AUC = 0.647, 95%C.I.(0.609, 0.686), P = 0.000001] and in both sex subgroups [men: AUC = 0.592, 95%C.I.(0.529, 654); P = 0.004; women: AUC = 0.758, 95%C.I.(0.711, 0.806), P < 0.000001], even there were sex-related differences in the best cut-off values (5.5 mg/dL for men; 4.2 mg/dL for women). Prospectively, SUA appeared predictive of middle-term (4-year) MetS incidence in the whole population (AUC = 0.604, 95%C.I.[0.518, 0.690], P = 0.029, best cut-off value = 4.7 mg/dL) and in the female group (AUC = 0,641, 95%C.I.[0.519, 0.762], P = 0.039, best cut-off value = 3.9 mg/dL) though not in the male one (P > 0.05). In conclusion, in our cohort, SUA is a frequent component of MetS, other than a middle-term predictor of newly diagnosed MetS in older women.

## Introduction

Currently evidence shows that subjects with high serum uric acid (SUA) levels have increased cardiovascular (CV) morbidity and mortality rates rather than the normouricemic ones^1^. However, it is controversial whether hyperuricemia is an independent risk factor for cardiovascular diseases (CVDs) developing or only a confounding one^2^, since many confirmed CVDs risk factors are per se associated with increased SUA^3–6^. Lacking a definitively proven causal association, a relationship of reverse causality has been also taken into account, whereby preclinical atherosclerosis could lead to higher levels of uric acid before a diagnosis of ischaemic heart disease^7^. There is also a growing evidence that SUA is involved in metabolic syndrome (MetS) incidence: several epidemiological studies showed a positive correlation between hyperuricemia and MetS^8^, which hyperuricemic subjects seem to more easily develop^9^. In fact, even though the exact biological mechanism is not yet known, uric acid seems to irreversibly react with nitric oxide (NO), disabling it and leading to endothelial dysfunction and, consequently, promoting the development of hypertension and MetS^10^. At the same time, NO has a well-known role in the insulin resistance and its shortage reduces the blood flow to the tissues sensitive to insulin, resulting in blocking of the insulin action^11,12^.

The aim of our study was at verifying if in a sample of overall healthy older subjects there is a correlation between SUA levels and MetS prevalence and middle-term (4-year) incidence. Moreover, we tried to identify SUA cut-off values, which could be functional as a diagnosis parameter of MetS in addition to the traditional ones.

## Material and Methods

The Brisighella Heart Study (BHS) is a prospective, longitudinal population-based investigation involving 2939 randomly selected Caucasian subjects resident in the northern Italian rural town of Brisighella. The BHS cohort consists in 1491 men and 1448 women, at enrolment aged 14 to 84 years and free from cardiovascular disease. The study started in 1972 and is still ongoing. The town of Brisighella was originally selected as the site for the study because of the homogeneity of life-style among its residents, with a very low rate of migration. Subjects were clinically evaluated at baseline and every four years thereafter, by collecting an extensive amount of clinical and laboratory data.

The BHS protocol and its sub-studies, which are largely described elsewhere^13^, have been approved by the Ethical Board of the University of Bologna and all of the involved volunteers gave their signed informed consent to participate^14^. All methods were performed in accordance with the relevant guidelines and regulations.

Blood pressure (BP) measurements have been taken early in the morning, after a 10 minutes rest in a quiet room, while subjects were in the seated position and by the use of a validated oscillometric device, with a cuff of the appropriate size applied to the right upper arm. The mean value of three blood pressure readings (obtained at 1-minute intervals) was considered as the variable of the study.

Laboratory analyses were carried out by trained personnel according to standardized methods^15^. They included total cholesterol (TC), triglycerides (TG), high-density lipoprotein cholesterol (HDL-C), low-density lipoprotein cholesterol (LDL-C), lipoprotein(a) (Lp(a)), apolipoprotein A-1 (Apo A-1), apolipoprotein B-100 (Apo B-100), fasting plasma glucose (FPG), serum uric acid (SUA), creatinine phosphokinase (CPK), estimated glomerular filtration rate (eGFR), total bilirubin (TB), aspartate aminotransferase (ALT), alanine aminotransferase (AST) and gamma-glutamyl-transferase (GGT).

**Figure 1:**
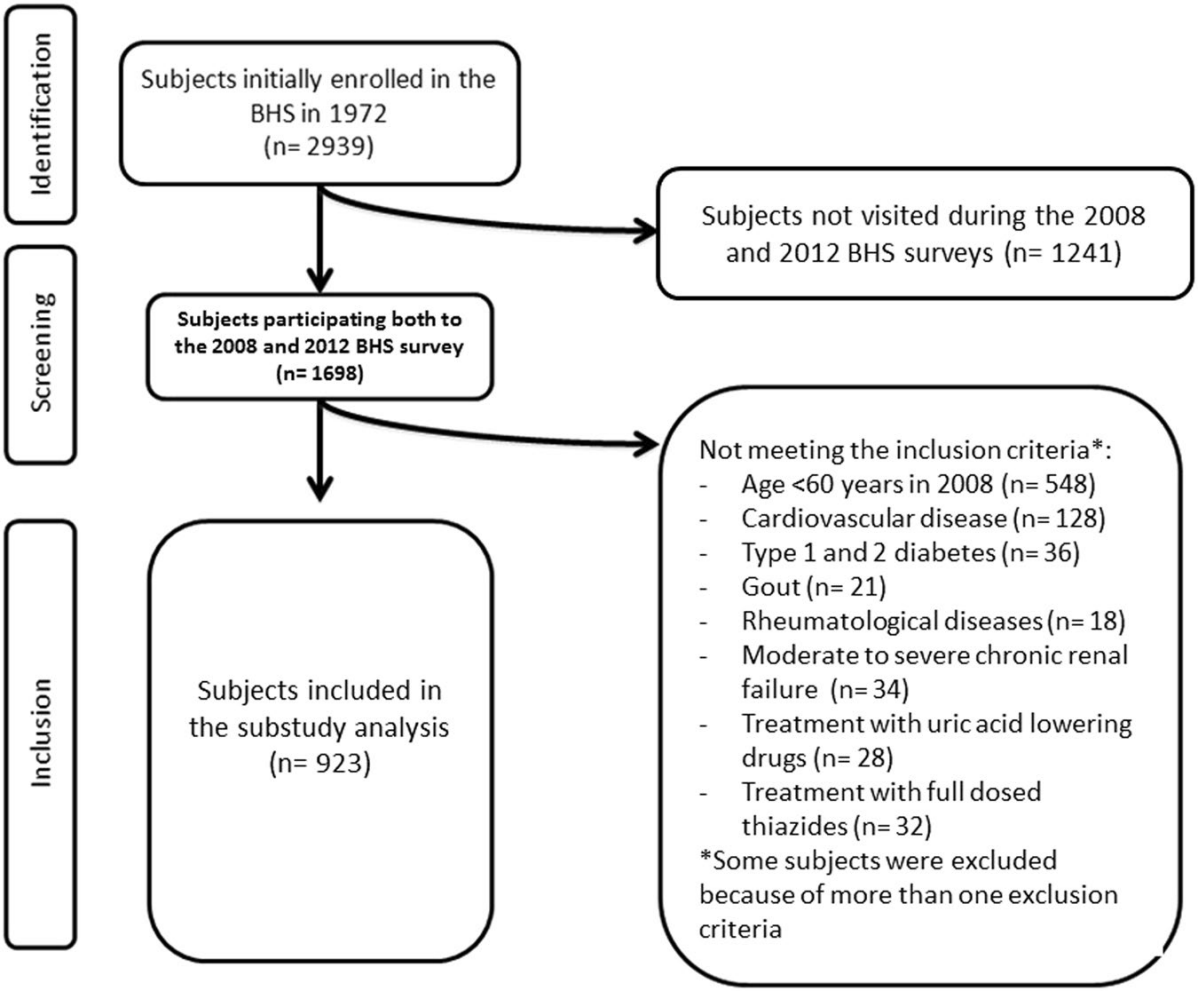
Flow-chart resuming the selection criteria applied to the full cohort to select the investigated subjects.

For the purpose of the present study, we selected 923 older volunteers (age >60 years; 434 males and 489 females) repeatedly visited during the 2008 and the 2012 population surveys. Subjects pharmacologically treated with SUA increasing drugs (namely high dosed thiazide diuretics) and SUA lowering drugs, affected by cardiovascular disease, type 1 and 2 diabetes, gout and other rheumatological diseases, and/or moderate to severe chronic renal failure were excluded from the present analysis (Fig. 1).

MetS was identified based on the ATPIII/IDF criteria^16^. The components were defined using the following ATPIII categorizations: 1) high blood pressure (BP ≥130/85 mmHg); 2) hypertriglyceridemia (TG ≥150 mg/dl), 3) low HDL-C (<40 mg/dl for men and <50 mg/dl for women); 4) hyperglycaemia (100 mg/dl >FPG), 5) waist circumference >102 cm for men and >88 cm for women (suggestive of high abdominal obesity). Subjects with at least three of the previous components were classified as having MetS.

We carried out a full descriptive analysis of all the considered variables. Descriptive values were always expressed as mean ± standard deviation (SD), because normally-distributedat the Kolmogorov–Smirnov normality test. Continuous parameters were compared by T-test for independent samples. First, we carried out a bivariate (Pearson) correlation for age, BMI, WC, SBP, DBP, TC, TG, HDL-C, LDL-C, Apo A-1, Apo B-100, AST, ALT, SUA, FPG and eGFR in overall population. Then, the analysis was repeated by sex. The risk estimate for MetS development was calculated and expressed as odds ratio (OR) and 95% confidence interval (95%C.I.). The receiver operating characteristic (ROC) curve analysis was firstly carried out using the MetS incidence at 2008 as state variable and the 2008’s SUA as test variable, in overall population and by sex. Secondly, in order to assess the predictive value of SUA on the 4-year MetS incidence, the previous analysis was repeated considering only those subjects who were not affected by MetS in 2008 (n = 669) but though they became in 2012. ROC curve was drawn using the MetS incidence in 2012 as state variable and SUA (as assessed in the 2008) as test variable. The analyses were performed in the general population and, then, separately by sex. Every analysis was two tailed. A P value less than 0.05 was regarded as statistically significant. Statistical analyses were performed through the SPSS 21.0 statistical software package (IBM Corporation, Armonk, NY, USA).

## Results

At the baseline, the population sample appeared to be mainly made up of middle-aged subjects, with a slightly tendency to overweight and hyperglycemia (Table 1).

**Table 1:** Main characteristics of the sample (2008), overall and sex-distributed, expressed as mean ± standard deviation.

Parameters	Whole population n = 923	Male n = 434	Female n = 489
Age (years)	72.0 ± 5.6	72.2 ± 5.6	71.8 ± 15,6
Body mass index (Kg/m^2^)	26.1 ± 4.2	26.5 ± 3.4	25.6 ± 4.7*
Waist Circumference (cm)	89.2 ± 12.1	93.2 ± 10.6	85.6 ± 12.3
Systolic blood pressure (mmHg)	131.6 ± 16.6	134.0 ± 15.4	129.4 ± 17.4^§^
Diastolic blood pressure (mmHg)	83.2 ± 10.4	85.6 ± 10.0	81.1 ± 10.5^§^
Total cholesterol (mg/dL)	205.1 ± 37.7	205.1 ± 38.2	205.1 ± 37.3
Triglycerides (mg/dL)	107.8 ± 66.7	116.1 ± 71.7	100.3 ± 61.0^§^
High-density lipoprotein cholesterol (mg/dL)	46.5 ± 9.7	43.8 ± 8.9	49.0 ± 9.8^§^
Low-density lipoprotein cholesterol (mg/dL)	137.3 ± 33.2	138.5 ± 33.9	136.2 ± 32.5
Apolipoprotein A1 (mg/dL)	144.0 ± 27.3	135.0 ± 25.3	152.1 ± 26.5^§^
Apolipoprotein B (mg/dL)	89.2 ± 20.7	92.0 ± 21.0	86.6 ± 20.1^§^
Fasting plasma glucose (mg/dL)	100.8 ± 9.6	103.7 ± 9.2	98.1 ± 9.2^§^
Serum uric acid (mg/dL)	4.8 ± 1.4	5.6 ± 1.3	4.1 ± 1.1^§^
Aspartate aminotransferase (U/L)	23.3 ± 9.1	24.8 ± 8.2	21.9 ± 9.7
Alanine aminotransferase (U/L)	24.3 ± 16.1	28.8 ± 18.8	20.3 ± 11.9
eGFR (mL/min/1.73 m^2^)	84.2 ± 5.8	85.2 ± 4.8	83.4 ± 6.5

eGFR, estimated glomerular filtration rate.

*P = 0.001 vs men; ^§^P < 0.001 vs man.

Considering the whole population sample, at the univariate analysis SUA positively correlated with age, BMI, waist circumference, SBP, DBP, TC, TG, LDL-C, Apo B-100, FPG, AST and ALT, while it was inversely associated with HDL-C, Apo A-1 and eGFR (Table 2). These results were robust in the subgroup analysis by sex (Table 2).

**Table 2:** Significant predictors of SUA in the whole population sample and by sex.

Parameters	Whole population		Male		Female	
	r	P	r	P	r	P
Age (years)	0.218	<0.001	0.132	0.006	0.368	<0.001
Body mass index (Kg/m^2^)	0.353	<0.001	0.323	<0.001	0.387	<0.001
Waist Circumference (cm)	0.500	<0.001	0.365	<0.001	0.468	<0.001
Systolic blood pressure (mmHg)	0.237	<0.001	0.133	0.006	0.256	<0.001
Diastolic blood pressure (mmHg)	0.196	<0.001	0.101	0.036	0.094	0.038
Total cholesterol (mg/dL)	0.161	<0.001	0.167	<0.001	0.214	<0.001
Triglycerides (mg/dL)	0.333	<0.001	0.358	<0.001	0.278	<0.001
High-density lipoprotein cholesterol (mg/dL)	−0.301	<0.001	−0.217	<0.001	−0.174	<0.001
Low-density lipoprotein cholesterol (mg/dL)	0.157	<0.001	0.129	0.007	0.201	<0.001
Apolipoprotein A1 (mg/dL)	−0.312	<0.001	−0.166	0.001	−0.186	<0.001
Apolipoprotein B (mg/dL)	0.231	<0.001	0.141	0.003	0.256	<0.001
Fasting plasma glucose (mg/dL)	0.345	<0.001	0.130	<0.001	0.343	<0.001
Aspartate aminotransferase (U/L)	0.224	<0.001	0.173	<0.001	0.168	<0.001
Alanine aminotransferase (U/L)	0.209	<0.001	0.085	NS	0.085	NS
eGFR (mL/min/1.73 m^2^)	−0.286	<0.001	−0.275	<0.001	−0.467	<0.001

eGFR, estimated glomerular filtration rate; NS, non significant.

Dividing the sample according to SUA mean (whole population = 4.8 mg/dL, men = 5.6 mg/dL, women = 4.1 mg/dL), MetS appeared to be more frequent for higher concentrations of SUA [OR = 2.12, 95%C.I.(1.55, 2.90) in men and OR = 2.69, 95%C.I.(1.91, 3.78) in women]. In the overall population, this tendency was confirmed when only the most severe forms of MetS were considered (with 4 to 5 risk factors) [OR = 2.59, 95%C.I.(1.58, 4.24)].

**Figure 2:**
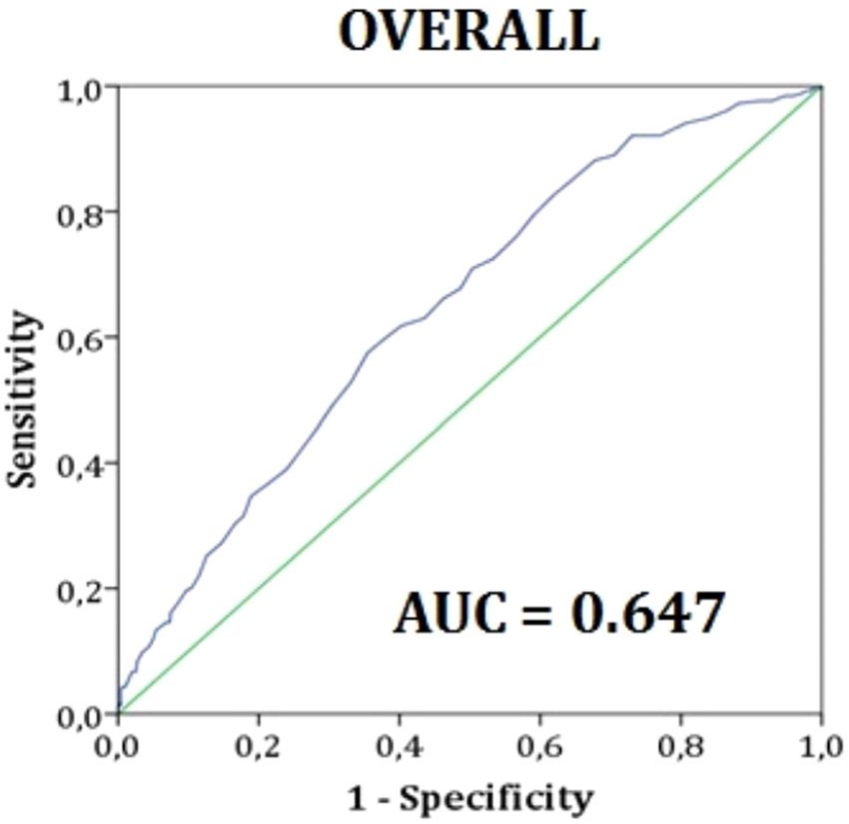
Receiver Operating Characteristic curve (ROC curve) constructed using serum uric acid concentrations (mg/dl) as test variable and metabolic syndrome as state variable in the general population. Data refer to the 2008’s population survey.

By drawing the ROC curves, SUA appeared a predictive test of MetS in the whole population [AUC = 0.647, 95%C.I.(0.609, 0.686), P < 0.001] and by sex [men: AUC = 0.592, 95%C.I.(0.529, 654), P = 0.004; women: AUC = 0.758, 95%C.I.(0.711, 0.806), P < 0.001] (Fig. 2), with sex-related differences in the best cut-off values (5.5 mg/dL for men; 4.2 mg/dL for women). Considering only the most severe forms of MetS (4 to 5 risk factors), this finding wasconfirmed in the overall population, with an AUC of 0.643, 95%C.I.(0.581, 0.705), P < 0.001 and a best cut-off point of 5.1 mg/dL.

Repeating prospectively the same analysis, SUA appeared again predictive of middle-term (4-year) MetS incidence in whole population [AUC = 0.604, 95%C.I.(0.518, 0.690), P = 0.029; with 4.7 mg/dL best cut-off value] and in the female group [AUC = 0641, 95%C.I.(0.519,0.762), P = 0.039; with 3.9 mg/dL best cut-off value], but not in the male one (P > 0.05).

## Discussion

Our findings largely agree with the available literature obtained in adult subjects^17,18^, which suggests that hyperuricemic subjects tend to develop MetS more frequently. SUA cut-off values currently considered in the gout treatment are higher than those proposed for the cardiovascular disease prevention^19^. Remarkably, our findings show that SUA levels associated with preclinical CVDs risk (as MetS is) are even lower.

Oxidative stress, induced by high levels of uric acid through the inhibition of endothelial NO bioavailability^20^, is supposed to result in a reduction of the endothelium-mediated vasodilatation, leading to a decrease of the blood flow in insulin target tissues. At skeletal muscle level, this lowers the insulin sensitivity, leading to hyperinsulinemia and insulin resistance on the long term. In adipose tissue, uric acid induces the expression of pro-inflammatory cytokines associated with insulin resistance^21^ and negatively modulates the activity of the nuclear receptor PPAR-γ, which acts as insulin sensitizer^22^. Furthermore, at the systemic levels, the induced endothelial dysfunction results in increasing the peripheral resistances, which also contribute to smooth muscle cell proliferation in the media^23^.

As negative modulator of the renal function, SUA may accelerate the progression of the hypertension, especially in the later stages of the natural history of hypertensive disease^24^. In this regard, several studies attested that a reduction in SUA levels with allopurinol treatment improves blood pressure values, validating his potential role in the pathogenesis of chronic hypertension^25^.

Numerous experimental evidence in humans and animal models, such as knockout mice for xanthine oxidoreductase^22^, support also that elevated levels of uric acid are also involved in the pathogenesis of obesity^26^, which is another diagnostic criteria for MetS. In fact, inducing the oxidative stress through NADPH oxidase activation, SUA inhibits the synthesis of adiponectin and generates oxidized lipids and inflammatory mediators such as monocyte chemoattractant protein-1 (MCP-1), determining a gain of weight^27^. Moreover, the visceral fat accumulation may also cause atherogenic dyslipidemia and increase hepatic SUA production. Actually, low SUA concentrations were just proved to improve the population’s heath status in general, also leading important positive economic effects^28^.

Thus, our findings strengthen for their part the Gerald Reaven’s hypothesis, who just included SUA in MetS definition decades ago^29^.

Certainly, the present study has some limitations. Primarily, the tight inclusion criteria to the analysis has reduced the number of eligible subjects. On the other hand, this has also cleaned the population sample from strong confounding factors. Then, the administered food-frequency questionnairecould not provide valuable data about the intake of dietary purines. However, the population dietary pattern is very well-known and believed to be stable because of the previous nutritional intervention^13^. Finally, we were not able to identify the hypoexcretors and the hyperproducers of uric acid. However,we excluded subjects with severely compromised renal function and results were adjusted for renal impairment, such as uric acid hyperproduction is supposed to underlie the metabolic adverse effects we observed.Then, the application of some exclusion criteria could have introduced a selection bias, but the included subjects remained representative of the general older Brisighella Heart Study cohort, being age and gender matched. By the way, our results are in total agreement with what observed in the unselected older cohort of the Progetto Veneto Anziani (Pro.V.A) study^30^, enrolled in an area relatively near to Brisighella. Finally, the accuracy of incidence data was obviously affected by the relatively brief period offollow-up. On the other hand, a number of confounding variables could have affectedfindings over a longerperiod.

In conclusion, relying on our data, hyperuricemia appears to be a highly prevalent component of MetS, especially in the most severe forms, as well as a risk factor for MetS developing, with cut-off values far lower than those considered dangerous nowadays in older subjects, and in particular in women.
